# How are substance use disorder treatment providers integrating harm reduction approaches into practice?

**DOI:** 10.21203/rs.3.rs-10502518/v1

**Published:** 2026-09-15

**Authors:** Morica Hutchison, Charles Neighbors, Sueun Hong, Ashly E Jordan, Pat Lincourt, Megan A. O’Grady

## Abstract

Background Harm reduction prioritizes reducing substance use-related harms (e.g., overdose) without requiring abstinence or reducing substance use to receive services. Substance use disorder (SUD) treatment has historically operated on an abstinence-based model, but in recent years there have been efforts to integrate harm reduction approaches into SUD treatment. However, little research has examined its acceptance among individuals who provide treatment in specialty SUD care settings; this study seeks to understand providers’ perceptions of harm reduction approaches including perceived and current benefits and challenges. Methods Semi-structured interviews were conducted with 21 individuals from 10 different New York SUD treatment programs. Framework analysis was used to code and analyze the data, which was guided by SAMHSA’s Harm Reduction Framework. Results We identified three overarching themes: 1) The landscape of harm reduction approaches is shifting in SUD treatment, 2) Challenges remain in implementing harm reduction approaches, and 3) Resources are needed to support the workforce in using harm reduction approaches. Seven sub-themes were also identified. Conclusions Overall, our findings point to a shift in SUD treatment programs’ views on integrating harm reduction approaches; staff support that these approaches can increase patient-centered and wholistic care, in addition to increasing treatment retention. However, ambivalence remains regarding its utility, noting challenges with facilitating mixed groups of patients and conflicting policies that impact their ability to provide informed, appropriate care. Findings point to staff and clinical supervisor training needs and system-level support that could support integration of harm reduction into specialty SUD treatment. Trial registration: NCT04632238

### Trial registration:

NCT04632238

## Background

Harm reduction is an approach to improve population health that prioritizes the ongoing minimization or prevention of substance use-related harms (e.g., overdose, HIV and hepatitis C virus transmission) over necessitating abstinence or reductions in substance use as a pre-condition to receive services(1–3). A harm reduction approach therefore is grounded in a lower-threshold healthcare delivery model aimed at engaging and retaining people in needed health services. At the same time, harm reduction-informed healthcare delivery also can include the provision of safer substance use supplies such as sterile syringes, other substance use equipment (e.g., cookers, cotton) and testing strips(4). Safer substance use supplies provided using a harm reduction approach have been an essential component of combined prevention, along with opioid use disorder (OUD) opioid agonist treatment (OAT; methadone and buprenorphine) and anti-viral treatment, to control and eliminate HIV and hepatitis C virus infection for decades(5–7).

Substance use disorder (SUD) treatment delivery models have historically relied on abstinence-based and 12-step models, including approaches that do not include medication-based addiction treatment (e.g., OAT). This approach stemmed from early SUD treatment models, heavily influenced by the Minnesota Model and Alcoholics Anonymous, which posited that addiction is a disease and people with addiction were biologically different. The model viewed individuals as not responsible for the onset of their addiction, but rather responsible for following the prescribed steps of a treatment program, which included abstinence from all substances(8, 9). However, in more recent years, there has been a shift that could signal a more fundamental integration of harm reduction approaches into SUD treatment, reflecting with recognition that harm reduction approaches may not be at odds with SUD treatment models. For example, treatment goals can include abstinence or reductions in substance depending on the individual's preference and needs. Further, the two approaches may work together as evidence suggests individuals who feel marginalized by traditional SUD treatment modalities are more willing to engage in harm reduction interventions(10). Therefore, leveraging the lower-threshold healthcare delivery model embedded in harm reduction approaches may facilitate earlier SUD treatment entry and lessen barriers to accessing and being retained in SUD treatment(11).

Within SUD treatment settings, harm reduction-oriented approaches may be evident by clinicians being more likely to consider patients' autonomy and create collaborative treatment goals, rather than using a one size fits all program with a singular focus on abstinence. Specific practices may include use of positive reinforcement(12), motivational interviewing to identify patients' goals related to substance use and providing treatment on the same day the person is enrolled in care (e.g., same-day buprenorphine prescribing(13–15)). Some states are providing guidance to SUD treatment programs on how to integrate this approach. For example, in New York State (NYS), the Office of Addiction Services and Supports (OASAS) released guidance in 2025 supporting the elimination of toxicology testing for punitive purposes and emphasizing the use of toxicology testing in a person-centered framework to support patients to meet their goals(16). Further, NYS OASAS released guidance in 2024 supporting an approach to ensure that the patient be retained in treatment including during periods of continued use or period of life challenges, specifically emphasizing avoiding discharging patients or discontinuing medication for "non-compliance" (i.e., missing appointments, continued substance use(17)).

Despite national and state efforts and preliminary evaluations demonstrating the benefits of integrating harm reduction approaches at a systems level, its acceptance among individuals who provide treatment for substance using populations remains uncertain given movement toward harm reduction in SUD treatment has been relatively recent. Most research on this topic has been conducted in primary care or independent medical settings, rather than specialty SUD treatment settings (hereafter referred to as 'SUD treatment settings'). Among staff in SUD treatment settings, the adoption of harm reduction approaches has been limited, despite increasingly positive attitudes. For example, Javadi and colleagues(18) reported that treatment professionals from SUD treatment settings generally had positive attitudes toward harm reduction approaches, with over 90% agreeing that MOUD and naloxone should be discussed as a treatment option, and over 80% agreeing that harm reduction strategies and distribution of naloxone are good for public health. Increased acceptance of harm reduction approaches was related to having been trained in harm reduction principles, specifically naloxone administration, and use of positive reinforcement to minimize shame and self-blame felt by patients. Barriers to acceptance of harm reduction approaches included the belief that abstinence should be the goal of treatment as well as not having enough information about naloxone(18).

Given the recent shifts in views on harm reduction and growing interest to integrate harm reduction approaches into SUD treatment settings, along with the limited literature describing SUD treatment providers' perceptions of this integration, we sought to understand NYS outpatient SUD treatment providers' perceptions of harm reduction approaches, including perceived and current benefits and challenges of using harm reduction approaches in their practice. Examining providers' perceptions and experiences can help inform how harm reduction approaches can be implemented effectively and sustainably, and point to potential areas of need for training or systems level interventions(19).

### Methods

## Procedures and Materials

The current study is a part of a larger stepped-wedge randomized controlled trial conducted as part of a government-academic partnership to test a multicomponent, clinic-level intervention, *Coaching for Addiction Recovery Enhancement (CARE)*, in various regions in NYS (Long Island, New York City and its suburbs, the Hudson Valley, and central New York) (Clinical Trial Number: NCT04632238; Registration date: November 12, 2020; clinicaltrials.gov). Inclusion criteria for the larger study were outpatient clinics licensed by the NYS OASAS with a minimum of 50 OUD patients per year. For further details on CARE, please see the protocol paper(20).

We used purposeful sampling to select staff who participated in the CARE intervention for interviews at the end of the 6-month active engagement portion of the study. Staff members were invited to participate via email and provided verbal consent. All staff who were invited to participate agreed. Interviews lasted ~ 45 minutes and were conducted and recorded via Zoom. Recordings were transcribed then de-identified and cleaned by the study team for analysis. There were two interviewers, both were PhD-level behavioral scientists with expertise in SUD treatment and qualitative research methods.

Interview questions covered a variety of topics related to the CARE intervention as well as clinical practice. Questions specific to harm reduction approaches were added for the final three cohorts of the CARE study because of significant efforts in the state at the time to incorporate harm reduction approaches into care and interest in how these approaches were being used. Questions covered how the interviewee defined harm reduction and the ways they incorporate it as it applies to their work with patients, any benefits or challenges of incorporating harm reduction approaches, and experiences with referring patients to external harm reduction services or programs. See Supplemental File 1 for further details on interview questions.

## Data Analysis

Framework analysis was used to code(21) and analyze the data. Atlas.ti(22) was used to manage the data. We analyzed data deductively using the 12 principles and 6 core practice areas of harm reduction according to SAMHSA's Harm Reduction Framework(23). SAMHSA outlines that these principles and practices give life to harm reduction approaches, initiatives, programs and services. Two researchers (MO, MH) read and familiarized themselves with all the interview data and created a codebook that aligned with the principles and practices in SAMHSA's framework. Due to overlaps, principles and practices collapsed into larger categories. For example, our *Overall safety* code combines original SAMHSA principles/practices of *safer settings, safer practices, and promotes safety*, whereas *Sustainable workforce, field and infrastructure* combine *sustainable workforce and field and sustainable infrastructure*, and *Whole personal healthcare access* combines *provide many pathways to well-being across the continuum of health and social care, safer transitions to care and safer access to healthcare*. The SAMHSA principle *Engage first* was dropped due to not being relevant to our project. Finally, we used inductive coding to add additional codes not in the SAMHSA framework, such as *Ambivalence* towards harm reduction approaches and beliefs about *Abstinence* regarding use of harm reduction approaches. See Supplemental File 2 for further details on the coding matrix used during analysis.

Following these decisions, two researchers (MO, MH) coded the same two transcripts, using the SAMHSA principles and practice areas(23), and met to discuss discrepancies and refine the codebook. One interview was identified as having no transcript, so it was removed from analyses. This process was repeated until the codebook was finalized (7 transcripts in total). Then, two researchers (MO, MH) independently coded the remaining 14 transcripts (7 each), reviewed each other's work, and met to come to a consensus on any discrepancy codes. After coding was complete, a matrix (SAMHSA principles/practices x interviewee) was created and reviewed to identify themes and data were examined in Atlas.ti to identify common co-codes. From the original 15 codes used during analysis, one sparsely used code collapsed into another code, leaving 14 total codes. During our next step of analysis, we identified three overarching themes within the coded data: 1) The landscape of harm reduction approaches is shifting in SUD treatment, 2) Challenges remain in implementing harm reduction approaches, and 3) Resources are needed to support the workforce in using harm reduction approaches.

Seven sub-themes which fit under three themes were also identified (described in detail below); see Fig. 1 for the final themes and sub-themes.

## Results

In total, 21 interviews from 10 different SUD treatment programs were conducted. Participants were on average 39 years old (range 20–65 years), mostly female (86%), White (62%), non-Hispanic (76%), and about a third each were counselors (38%), directors (29%) or other staff (29%; e.g., coordinator roles). Findings from the interviews were categorized under three main themes.

### Theme 1. The landscape of harm reduction approaches is shifting in SUD treatment

The first theme focused on how harm reduction approaches are integrated within clinical practice and organizational approaches. In general, staff found integration complex. One participant noted, *"harm reduction is a little complicated in this world that we live in, because we also went so hard from abstinence-based to harm-reduction, and it felt like it was overnight."* (Participant 03)

This perspective is highlighted in two sub-themes. The first focused on clinical practice changes due to integration into care. Staff believed it was important for patients to drive their treatment goals to allow them to feel heard and empowered, as highlighted by one staff member who stated:

*We got to just remember that that's what person-centered care is; and we can still have*

### clinical recommendations and referrals, and we can still stick to those. That doesn't mean

*we're letting people run rampantly wild, but we meet them where they're at, in the hopes of*

*maybe that's the direction they're going in. But we're not forcing it. We can't force it.*

(Participant 03)

Staff also indicated that it was their role to have an attitude of non-judgement towards patients' goals, even if they didn't align with the staff's own goals. One staff member noted:

*We've had different people just see that, that we care, that they're doing okay even if they're*

*relaxing or if they're still using…we still wanna see you. Even if you just used last night, we*

*still wanna see you. We're not gonna judge you. It's okay. It's not okay to be using, but it's*

*okay to come get help. And it's okay to come see us. And we're glad to see you and all of*

*that.* (Participant 01)

Staff highlighted the changing policies and procedures that impact clinical care provided, and how more changes are needed to fully embrace a harm reduction approach to clinic policies and offerings. Many staff suggested that there was a stronger emphasis on providing MOUD as part of a harm reduction approach:

*I know there's some clinics that if the client's actively using more as a punishment of you're*

*not being dosed today, we can't give you your medication, here, we don't do that… just the*

## idea of not taking away "take homes" (methadone) because they're actively using or they're

*not showing up, that kind of thing. It's at least giving them their medication. We know that*

*there's a chance that they're taking it if they have it. I know the clean needles, that kind of*

*thing. We don't actually do that here. I know we've been talking about it, supplying the clean*

### needles, needle exchange, that kind of thing. But more along the lines of that, the

*medication piece is the biggest part of it, not withholding medication.* (Participant 04)

Challenges were noted in changing clinical practice to be more patient-centered, such as staff needing to amend their perspective on what they wanted the patient to accomplish compared to what the patient wants to accomplish for themselves to improve patient retention:

*Trying to really work with somebody on what it is they wanna accomplish; not what I want*

*them to accomplish. I hope we build a better rapport with that patient and have them last*

*longer. Complete a program, feel good and reach the goals that they've set themselves.*

(Participant 13)

Similarly, staff indicated new perspectives about what harm reduction approaches means in terms of patient treatment: *"I think it's a door...I used to think it's, "Oh, my. You're condoning these patients. You've got them shooting drugs," and this and that. No. No. No. No. No. No. It's a door."* (Participant 06)

The second sub-theme focused on organizational approaches to clinical services, as well as cultivating relationships with external providers and the community. For example, using a harm reduction approach allowed for more focus on whole person healthcare, such as providing resources (Narcan, fentanyl test strips), providing support (e.g., referrals to peer services), and community-based education for service providers on how to integrate, rather than a narrower focus on substance use. Many programs have focused on forming relationships among community-based providers to create an easy referral process for additional support and offered community-based services such as Narcan training or educational sessions with families and criminal/legal entities about harm reduction approaches and their benefits. For example, one staff reported, *"We offer Narcan, and we offer Narcan training. And we have peer advocates that are available at any point. And we have other community resources and things that we provide all of that information for our clients."* (Participant 01), while another stated:

*We also do lots of education with other local providers and other entities in the community*

### like probation, parole, DSS. Kind of having conversations around harm reduction and how

*that supports our clients and our role versus their role. So, we do lots of education with the*

*other people that our clients may come in contact with. And I think that it's been embraced,*

*and I think that clients are mostly realistic about harm reduction and what that looks like for*

*them and for their certain situation.* (Participant 20)

Despite these positive efforts, staff reported challenges in terms of addressing external aspects to patient care, such as transportation barriers, and not having in-house services with variation in patient follow-through, leading to some organizations hiring harm reduction specialist staff. For example, one staff indicated:

*Because we do work with a lot of clients who don't wanna stop using, but they want it to*

*impact their job or they're in family court or they have those other external reasons why they*

*probably should stop. And a lot of time I've noticed that when I refer them to these external*

*places, they don't do a good job at following through. So, with us being able to hire on a*

## harm reduction specialist we're hoping to make it so that there's more follow-through. Then

*we can actually really address these things more in house so they're not getting lost in the*

*weeds.* (Participant 08)

### Theme 2. Challenges remain in implementing harm reduction in SUD treatment

Despite positive progress toward harm reduction approaches in SUD treatment, staff perspectives on harm reduction approaches are not straightforward. Two sub-themes of challenges were identified. The first was staff ambivalence towards incorporating harm reduction approaches in both clinical practice and treatment philosophies. On one hand, many staff acknowledged some benefits of moving from abstinence-based to harm reduction focused approaches, including being able to provide better patient-centered care and improve retention:

*We have always been a complete abstinence based program. And, I do think in the past, it*

*was very black and white. It was either you’re doing this or you’re not in the program. And, I*

*do think that we would lose a lot of clients. We would lose a lot of clients. And now, we see*

*clients stay longer. They continue to receive the support that they need. And often, we do*

### see a couple of clients eventually go complete abstinence, which is great. Ultimately, being

*there for clients in whichever way we can be is the goal here.* (Participant 17)

On the other hand, staff reported questioning whether harm reduction approaches are beneficial for patients, feeling that harm reduction condoned continued use and provided a false sense of security. Ultimately, participants reported that it could be a struggle to balance patient-centered care while ensuring the patient remains safe. For example, one staff reported:

*Even with people who really strongly believe in harm reduction and person-centered care,*

*everybody is on a spectrum of it, and we will constantly run into… where does person*

### centered care and harm reduction become more of a danger to a patient than not?

(Participant 03)

While another said:

### Well, you're always going to get people who feel that harm reduction is just somebody's

*excuse to still get high. Somebody's on methadone because they just wanna still get high.*

*Or somebody wants to just smoke weed. So, that's an excuse to just smoke weed. It's not*

*really just a harm reduction thing or they're not just using it therapeutically when really, it's now considered a harm reduction approach. So, the challenge is trying to help somebody understand that it's now utilized as a form of helping somebody really stay alive. And that's what it is. It's an alternative to a more dangerous drug. That's what it is.* (Participant 05)

Further, staff reported difficulty managing their own feelings around patients continued use.

*So, on paper, harm reduction looks really nice. On application, you're dealing with clients who have no ability to moderate what they're doing or how much they're consuming. If they're going in, they're going all in. So, if they're gonna have one tall boy, they're probably gonna have eight to 10 tall boys. Maybe some days they have five and that gives them the small sense of confidence and self-efficacy, but then the next day they're back up to 10 and that just creates this depression, this drop, they don't think they can do it, they can't believe in themselves. So, harm reduction looks nice, but in application it doesn't really work all that well because of the population we're working with. The whole definition of addiction is that they can't control it. So, if we're trying to give them this idea that maybe they can control it, we're completely ignoring what it means to have an addiction.* (Participant 08)

The second sub-theme focused on the impact of staff clinical training and perceptions on the benefit of abstinence-only treatment. Specifically, we saw a mix of "old and new school" perspectives from staff, often based on time in the field or training. Many of the newer staff had embraced a harm reduction approach, while those from the 'old school' perspective struggled, with one staff reporting:

*But again, I guess, harm reduction, it's definitely new for me. I've been in the field almost 18 years. So, when I first started it was definitely an abstinence-based model. And then when you look at the definition of addiction according to ASAM and the DSM5 they identify it as a*

### brain disease. So, regardless of the amount of substance going in, it's still affecting your

*brain. So, I mean I guess I have some personal opinion that it might be counterproductive*

*according to science.* (Participant 10)

Another common thought was whether harm reduction approaches are real recovery or slows patient progress, as one staff member stated:

*Or going completely cold turkey off of all the substances. Or maybe they recognize that,*

*"Okay. I literally can't drink. I can't smoke. I can't do any of these things responsibly." And*

*that's gonna send them to AA. They start to explore these other things. But with the harm*

*reduction it just delays almost the inevitable.* (Participant 08)

### Theme 3. Resources are needed to support the workforce in using harm reduction approaches

The final theme focused on the support needed to address challenges within the workforce (e.g., hiring practices, staff safety), clinical skills (e.g., ongoing training, adjusting preconceived notions about harm reduction approaches), safe supply resources needed, and clinical supervision focusing on harm reduction approaches. Three sub-themes were identified, the first focused on resources needed to support the safety of patients. Staff reported resources (e.g., supplies) needed by programs for safe use, with one staff stating:

*We have xylazine test strips. We have fentanyl test strips. We offer naloxone. It's a part of our*

### initial assessment. When a client comes in, they're offered Narcan training; and even

*ongoing, we offer it. We give strips out constantly. We have them right at the front, so, if*

*anybody is interested, they can just grab them. Yeah, we're not stingy about the resources.*

(Participant 16)

They also needed more support and clinical training in how to support patients who continue to use substances, particularly to counter the false sense of security that they felt patients had. For example, one staff noted:

*On the other hand, it gave them this false sense of safety in the fact that they weren't going*

*to overdose because then they think, "Oh, well, I get it from this supplier. And because this suppliers never had fentanyl in it according to my test strips, I'm gonna stop testing." And I've seen this with a client. So, they stop testing and, all of a sudden, they're in the hospital.*

**And they're in a hospital because they stopped testing because they were given a false**

*sense of safety that was coming from these test strips that was coming from this dealer. It starts to backtrack.* (Participant 08)

Tied within this subtheme were discussions around the need for clinical strategies to manage mixed groups (i.e., having patients who are both abstinent and not). For example:

*The second biggest challenge that I see is harm reduction in the group setting is really hard,*

**because of just the combination you have of people believe strongly in abstinence. It's**

**challenging to balance that in the appropriate way, to keep patients from almost triggering**

*each other. We have guys all the time that talk about smoking marijuana in group, and you're like, "I don't want to tell you what to say in group, but you've got to remember that you talking about that is so triggering to the guy next to you who's not." So, the balance of those things is really hard.* (Participant 03)

The second sub-theme focused on support needed to work towards system change to improve use of harm reduction approaches. This included the need for better alignment between the criminal legal system and SUD treatment system and better strategies to support patients who are mandated populations or involved with other criminal legal system entities (e.g., probation/parole, drug courts). Multiple staff indicated challenges regarding probation and parole agencies, specifically the competing rules and regulations of abstinence-based versus harm reduction-based approaches:

*The only time we really run into a significant issue is if they have a legal entity such as*

**probation, drug courts, things like that, obviously they're not recognizing a harm reduction**

*model. So, we kinda have to work with the client. Like, "You have these set of rules through your legal entities that say you need to remain abstinent from all substances. How can we assist you in getting to that point?* (Participant 10)

The final sub-theme focused on the need to support a sustainable workforce, field and infrastructure. Discussions focused on challenges in staffing, particularly for those from abstinent-only perspectives moving to integrate harm reduction approaches, and the need to reframe from years of training:

*There are a lot of folks who have been in this work a really long time, who are themselves in*

**recovery, and from the 12-step world, which is very abstinence focused. There's a lot of**

**resistance to harm reduction from those folks. There's an idea that it's not "real recovery."**

**So, the biggest resistance honestly comes from, I think, the practitioners themselves and**

*sometimes the 12-step world.* (Participant 19)

Some staff reported no issues in this area as most of their staff were newer in the field and trained in harm reduction approaches from the start, whereas others indicated significant resistance from staff. In terms of staff training on harm reduction approaches, both past and ongoing, a few noted the free training and webinars related to harm reduction and patient-centered care offered by NY OASAS as beneficial but wanted more training around strategies on how to implement harm reduction approaches in real world settings with examples from the field and support from those already implementing this within their programs. One staff member noted:

*OASAS has been really helpful. I know they have a lot of free trainings on their website and stuff that we use a lot. We have in-house trainings as well. Like I said, I'm new to this field, so I'm still learning. Every day is different. So, it has been helpful.* (Participant 04)

While another indicated:

*We have enough people who have been using harm reduction as a model, that if they were interested to be able to get them to conduct some of these trainings…and you have a few minutes from someone who actually experienced it talking about what it was like for them,*

*how difficult it was to make the transition, and then sort of where they're at now. I think that's a lot more moving for people, because it's a real-life individual who could say, "Yeah, no, I didn't think this would work; and I was just kind of at the end of my ropes, and I tried it out, and now I can travel.* (Participant 16)

Finally, staff indicated the importance of clinical and group supervision within the application of harm reduction approaches:

*Clinical supervision is really important. The group supervision, to the staffing. You know, being able to staff cases and come together and try to come up with a solution or a way to try to help a patient in a different way…But also, feeling supported. So, there's patients that really may not be appropriate for a certain program, and the counselor may feel that they're*

**not appropriate for different reasons. Legitimate reasons. And feeling supported and making**

*a different referral or a different decision that might be more appropriate for that patient; more beneficial.* (Participant 13)

Staff reported wanting additional support to train supervisors on harm reduction approaches and best practices to share with staff during supervision. Support is also needed to hire staff dedicated to harm reduction programming within clinics. For example, one individual stated:

**The biggest challenge? Staffing. Funds. Because I've spoken to the project director…our**

**harm reduction program is currently a grant program. And he's had conversations in which**

*regards to – he's trying to hire staff, and then he has his staff going out into the community, and so forth. And just the work that they do on a day-to-day basis, more is needed. And I've had this conversation with him in regards to, "You're a grant program now. You're gonna be a program because it's needed.* (Participant 18)

### Discussion

The current study examined SUD treatment staff perspectives and beliefs regarding harm reduction approaches, how it is integrated into their clinical work with patients, and the benefits and challenges of utilizing harm reduction approaches. Given the limited adoption of harm reduction approaches and the paucity of research on SUD treatment providers' integration, this study fills an important gap in our knowledge. Overall, staff indicated that the landscape is changing, such that SUD treatment clinics are moving away from abstinence-based models and toward more individualized care that allows patients to set their own goals; but challenges remain (e.g., ongoing ambivalence toward harm reduction approaches among some staff). Further, we found that system-level support is needed to create a sustainable workforce that prioritizes whole-person healthcare outcomes with a focus beyond just substance use (e.g., life satisfaction, employment).

Findings supported that the shifting landscape toward harm reduction approaches within SUD treatment has resulted in the need to change clinical practice and organizational approaches. For SUD providers, harm reduction is not just providing supplies (e.g., clean needles, safe use kits); it is an important avenue to provide person-centered and wholistic care, with a chance to improve access and retention in treatment. This has required a change in attitudes and practices from staff in terms of goal setting with patients and utilizing non-judgmental approaches. Organizations have also needed to cultivate external relationships with community-based services to facilitate referrals to whole-person care. There does remain a good deal of ambivalence and skepticism despite changing attitudes, particularly for staff who have been in the field for some time. There is an opportunity to better support clinician skills in using harm reduction approaches via training and clinical practice resources. For example, such training could focus on how to support the patient's treatment goal preferences that may conflict with providers' preferences.

One of the biggest challenges in using harm reduction approaches was managing group counseling sessions with patients who were still using substances, mixed with patients who were abstinent. Staff reported challenges in how to best manage group dynamics to create a welcoming, safe environment without triggering patients at different stages of recovery. Training and support should be prioritized in this area. For example, training on the management of groups of mixed patients may follow ASAM strategies: removing the disruptive patient from groups and providing more individualized treatment services while the issue is being addressed, providing additional services or referrals to address underlying drivers of substance use or barriers to recovery, offering groups tailored to those who are in the contemplative or precontemplative stages of change, debriefing following any significant disruptions when it is safe to do so, and communicating group expectations regularly (e.g., respect, community safety, conflict deescalation strategies)(24). ASAM urges staff to consider alternative clinical options prior to administrative discharge, like policies set forth in NY state, and suggest training focused on helping staff to manage these therapeutic situations safely and effectively.

Further, providing education on safe use practices and offering resources that may extend beyond what has previously been offered in SUD treatment settings is needed. Programs require stable sources of supplies (e.g., Naloxone) and training on how to increase patients' consistent use of these. While evidence suggests that providing harm reduction supplies does not increase crime or drug use in communities where they are available, and can reduce overdose rates and health risks(25), staff report that challenges persist in patient's consistent and appropriate use of them. One such strategy to support staff in this area may be to provide Motivational Interviewing training focused on strengthening the patient's own motivation and confidence to protect their health, to help reinforce their ability to make safer choices and see the gap between their current practices and personal goals. Previous literature echoes these challenges and suggests the importance of supporting staff to set boundaries with patients, access to resources and knowledge to support patient needs, and the importance of hiring staff whose beliefs align with clinic priorities of harm reduction approaches(26).

Staff also need support to more effectively work with mandated and/or criminal/legal involved-patients within the parameters of a harm reduction approach. Evidence suggests that local law enforcement practices create additional barriers that can hinder the implementation of harm reduction approaches due to a lack of widespread adoption, and varying policies that influence stigma related to SUD treatment(27, 28). While we note this is a longstanding challenge, it is imperative that the criminal/legal system and SUD treatment system work together to form a shared understanding of harm reduction approaches, such as through convening work groups to facilitate policy change around patients within both systems' ability to utilize harm reduction approaches without punitive repercussions.

Our findings point to the support needed to ensure that harm reduction approaches are seamlessly implemented, including how to maintain safety for patients and staff, strategies to create system level changes to support harm reduction, and how to create a sustainable workforce. One focal aspect discussed by staff was clinical supervision. Staff felt clinical supervision is often not adequate in the new system of integrated harm reduction approaches. Training and resources specific to clinical supervisors could help support system level change. For example, clinical supervisors may benefit from specific training focus on helping staff to: integrate harm reduction approaches with patients, create a therapeutic environment for all patients regardless of substance use status, and to address special circumstances when using harm reduction approaches with patients involved in the criminal-legal system(11).

While making training readily available is a good start, it is imperative to move beyond this to provide implementation examples, support and guidance on how to integrate harm reduction approaches in real world settings, paired with examples from lived experience. Providing comprehensive training from individuals with lived experience can help to shape the curriculum, language and examples used to be inclusive and relevant to the community, and to equip staff with the skills and knowledge necessary to support the unique needs and experiences of individuals with continued use. Relatedly, in Oregon, state-level harm reduction efforts have been recently implemented, focusing on three main goals: 1) promote provider training and education, 2) expand naloxone access, and 3) promote MOUD[10]. Preliminary evaluation of these efforts has demonstrated improved workforce knowledge related to the three goals, a 346% increase in naloxone prescription claims from the first quarter of 2019 to the first quarter of 2022, and consistent increases in the number of providers able to prescribe buprenorphine since 2017.

Additional examples from implementation efforts are needed to understand provider knowledge about and uptake of harm reduction-oriented treatment approaches, while considering the challenges and benefits to utilizing such an approach in patient care.

Finally, integration of harm reduction approaches into SUD treatment could be improved by continued support for the workforce at a more systemic level. For example, hiring specific harm reduction staff to support the treatment team. However, this would require financial support or billable strategies to make this practice sustainable. Further, programs with harm reduction approaches should focus on hiring staff who are open to integration into their practice. For instance, one study found clinics were more likely to utilize harm reduction approaches if they hired staff and providers willing to adopt a low-threshold, stigma-free approach to care, conducted ongoing harm reduction trainings both internal and external to the organization, with a focus on undoing previous training that was at odds with a harm reduction approach, established a referral network with external providers in the community, and offered flexible services to patients (e.g., telehealth appointments).(26) However, systems levels supports will be needed to ensure that the workforce is well educated and skilled in order to require this as a hiring policy.

## Limitations

The study is not without its limitations. Participants were from SUD treatment programs in NY State, a state which has had some efforts to improve use of harm reduction approaches, thus limiting the generalizability to other states, especially those that have not made efforts around harm reduction. Further, because this was part of a larger study, we did not sample based on harm-reduction views; it is possible that if we specifically sampled individuals with both negative and positive perceptions of harm reduction approaches that our findings could differ.

## Conclusions

Overall, our findings point to a shift in SUD treatment programs' views on integrating harm reduction approaches into patient care. Staff support the belief that harm reduction approaches can increase patient-centered and wholistic care, in addition to increasing treatment retention. However, some staff remain ambivalent about the utility of harm reduction approaches in real world settings, noting challenges with facilitating mixed groups of patients and conflicting policies that impact their ability to provide informed, appropriate care. Previous research suggests barriers to utilizing harm reduction approaches include the referral and relationships with external providers, including penalization for missed appointments, the individuality of patient needs, availability of resources, and hiring the right kind of people to work at the clinic(26). Given that limited research has focused on SUD treatment staff perspectives and utilization of harm reduction approaches, we echo the previous research, and also believe our findings point to next steps in terms of staff and clinical supervisor training needs, and strategies of how to create a shared understanding of harm reduction approaches among SUD treatment providers, community-based providers and the criminal/legal system. Supporting staff via training and policy change will aid in the continued positive acceptance of harm reduction approaches within SUD treatment to improve patient care, treatment retention and outcomes.

**Supplemental File 1.** Harm Reduction Interview Questions

1) **How would you define harm reduction as it applies to your work with clients?**

a. In what ways, if any, have you incorporated harm reduction principles into your work with clients?

b. What has been your experience incorporating harm reduction principles into your clinical work?

c. What do you see as a benefit of incorporating harm reduction principles? Challenge?

d. Have you referred clients to external harm reduction services/programs? If so, please describe your experience in doing this.

**Supplemental File 2.** Coding Matrix

| Code Name | SAMHSA Definition | Contextual Definition | Examples |
| --- | --- | --- | --- |
| ***Theme 1: The Shifting Landscape of Harm Reduction*** | | | |
| **Sub Theme 1: Changes in Clinical Practice** | | | |
| Respect Autonomy | Each individual is different. It is important to meet people where they are, and for people to lead their own individual journey. Harm reduction approaches, initiatives, programs, and services value and support the dignity, personal freedom, autonomy, self-determination, voice, and decision making of PWUD. | Clinicians discuss meeting patients where they are at and that patients' need to lead their own individual journey in terms of personal freedom, autonomy, and decision making of treatment goals and progress. | So, obviously, for me, harm reduction, the biggest thing that I think of – the instant thing that I think of when I think of harm reduction – is just saving people's lives, whether that is with opiates or… with alcohol, we do harm reduction a lot, of just reducing use, and using safely, and meeting people where they're at, as far as what their goals are. 34072_CR0111_10_2023CH (C4) |
| Practice acceptance and hospitality | Love, trust, and connection are important in harm reduction work. Harm reduction approaches, initiatives, programs, and services hold space for people who are at greatest risk for marginalization and discrimination. These elements emphasize trusting relationships and meaningful connections and understand that this is an important way to motivate people to find personal success and to feel less isolated. | Clinician discussion of holding space for people who are at highest acuity, or those coming to treatment who normally would not have come or remained in treatment due to ability to practice and offer harm reduction to build trusting relationships and meaningful connections to motivate people to find success and feel less isolated. | Having such a larger support system for people, adding an extra person or people or a whole team that someone can call on and rely on and reach out to. And so that they know that they have a safe place and safe people to go to even if they've relapsed or if they're in a tough place. IF they're having difficulty communicating, we're here. We're here to help and we can figure out what their needs are. So, I feel like that is definitely big and sometimes monumental for people, especially if they've just gotten out of jail and they don't know how to deal with the real world because they've been in prison for so long and different things like that, so helping them just navigate life. NC03-071624-Ct (C6) |
| Provide support | Harm reduction approaches, initiatives, programs, and services provide information and | Clinician discussion of how they provide information and support without | And so, for us, the harm reduction is having the conversations with the individuals. It's talking to |
| Notes. SAMHSA guidelines link: Harm Reduction Framework; PWUD = person who uses drugs ; 'overall safety' combines the following codes: safer settings, safer practices, and promote safety ; 'Sustainable workforce, field and infrastructure' combines sustainable workforce and field and sustainable infrastructure' ; dropped 'engage first' as it was not relevant to our project; 'whole person healthcare access' combines the following codes: Provide many pathways to well-being across the continuum of health and social care, safer transitions to care and safer access to healthcare. | | | |

| Code Name | SAMHSA Definition | Contextual Definition | Examples |
| --- | --- | --- | --- |
| ***Theme 1: The Shifting Landscape of Harm Reduction*** | | | |
| **Sub Theme 1: Changes in Clinical Practice** | | | |
| | support without judgment, in a manner that is non-punitive, compassionate, humanistic, and empathetic. Peer-led services enhance and support individual positive change and recovery; and peer-led leadership leads to better outcomes. | judgement, in non-punitive, compassionate, humanistic and empathetic manners, including supporting individuals positive change and recovery. | clients about what does appropriate use look like. Impacts. But I think it's also having conversations with individuals that are identifying as wanting harm reduction or really feeling like that's more appropriate if they do have that mandated option to having those conversations. Or like, "We're here to support you. And part of that supporting you is also let's talk about what potential consequences may come from the harm reduction, continuing to use, and things like that." sc0204_11_2023ct (C5) |
| Assist, not direct | Harm reduction approaches, initiatives, programs, and services support people on their journey towards positive change, as they define it. Support is based on what PWUD identify as their needs and goals (not what programs think they need), offering people tools to thrive. | Discussions of how clinicians support people on their journey towards positive change, as the patient defines their needs and goals, offering people tools to thrive. | "And so, harm reduction looks different for each individual. And so, getting the staff to understand that we're not forcing a patient to stop, but we're giving them educational support, coping skills, etc. Resources so that while they're in this process, while they're in this transition, they can make the decision to how they wanna go and be there to support them along the way." vip0211_13_2023ch (C4) |
| **Sub Theme 2: Organizational Approaches to Harm Reduction** | | | |
| Connect with community | Positive connections with community, including family members (biological or chosen) are an important part of well-being. Community members often assist loved ones with safety, risk reduction, or | Discussion of positive connections with community, including family or other social supports, strategies to include these supports in services, and their influence on safety, | Yes. Absolutely. So, every time a client is discharged, we make sure that they are – we at least offer it. Not all clients want it, but we at least offer to train them and provide them with a naloxone kit. And then we also have a family program. So, oftentimes when the family comes in, we will |

Notes. SAMHSA guidelines link: Harm Reduction Framework; PWUD = person who uses drugs ; 'overall safety' combines the following codes: safer settings, safer practices, and promote safety ; 'Sustainable workforce, field and infrastructure' combines sustainable workforce and field and sustainable infrastructure' ; dropped 'engage first' as it was not relevant to our project; 'whole person healthcare access' combines the following codes: Provide many pathways to well-being across the continuum of health and social care, safer transitions to care and safer access to healthcare.

| Code Name | SAMHSA Definition | Contextual Definition | Examples |
|---|---|---|---|
| ***Theme 1: The Shifting Landscape of Harm Reduction*** | | | |
| **Sub Theme 1: Changes in Clinical Practice** | | | |
| | overdose response. When possible, harm reduction initiatives, programs, and services support families in expanding and deepening their strategies for love and support; and include families in services, with the explicit permission of the individual. | risk reduction and overdose response. | offer it to the family while their loved one's still actively using. So, we make sure that it gets kind of on both sides. 34072_rs0111_13_2023ch (C4) |
| Whole-person healthcare access | Harm reduction can and should happen across the full continuum of care, meeting whole-person health and social needs. In networking with other providers, harm reduction initiatives, programs, and services work to build relationships and trust with care partners that embrace supporting principles. Organizations practicing harm reduction utilize education and encourage policies that facilitate interconnectedness between all parties. Ensuring connections and access to person-centered and non-stigmatizing healthcare that is trauma informed (e.g., Low-barrier treatment services; FDA-approved medications; Flexible provision of services; | Discussion of efforts to use person-centered and non-stigmatizing care that meet the whole person's health and social needs and is trauma informed, including referrals or making connections with other providers or patients to build relationships that support harm reduction principles, FDA approved medications, low-barrier treatment services (whole-person approach and rapid re-initiation, flexible provision of services, nonpunitive care, mobile access and take-home medications; telehealth services in patients preferred language, warm hand-off to/from emergency department programs, medication | I actually used to work with our local needle exchange. I actually was their peer service supervisor. So, we definitely collaborate all of the time. We understand that we're not a one stop shop. And we do have people and we have fluxes where people will come in and want safer use supplies. So, we have a partnership with [Project name] where we can call them directly and they will come and do deliveries, or we'll get them involved. That way they have a warm handoff with that person. For our agency, we also do a lot of outreach. And so, our needle exchange then for example, goes right up the road to one of the sites. 34072_NC02-071124-CT (C6) |
| Notes. SAMHSA guidelines link: Harm Reduction Framework; PWUD = person who uses drugs ; 'overall safety' combines the following codes: safer settings, safer practices, and promote safety ; 'Sustainable workforce, field and infrastructure' combines sustainable workforce and field and sustainable infrastructure' ; dropped 'engage first' as it was not relevant to our project; 'whole person healthcare access' combines the following codes: Provide many pathways to well-being across the continuum of health and social care, safer transitions to care and safer access to healthcare. | | | |

| Code Name | SAMHSA Definition | Contextual Definition | Examples |
|---|---|---|---|
| ***Theme 1: The Shifting Landscape of Harm Reduction*** | | | |
| **Sub Theme 1: Changes in Clinical Practice** | | | |
| | Healthcare settings and providers are directly informed by harm reduction principles; Expand telehealth, while also addressing low technology literacy and enhancing access to other languages; Warm hand-off to and from emergency departments; Onsite or immediate referral to accessible nutritional assistance, clothing, temporary shelter, and housing; Seamless coordination of care for individuals leaving carceral settings) | access and treatment on-demand, clothing and housing, care coordination for individuals leaving carceral settings/treatment settings that don't offer medications | |
| Cultivate relationships | Relationships are of central importance to harm reduction. Harm reduction approaches, initiatives, programs, and services are relational, not transactional, and work to establish and support quality relationships between individuals, families, and communities. | Discussion about relational, not transactional, aspects of working with a client, including strategies to establish and support quality relationships between individuals, families and communities. | |
| Prioritize listening | Each community has its own unique story that can be the foundation for harm reduction work. When we listen deeply, we learn what matters. Harm reductionists engage in active listening — the act of inviting people to express themselves completely, recognizing | Discussion of engaging in active listening, inviting patients to express themselves completely, recognizing their own inherent biases, including tolerance of harm reduction for certain substances, with the intent of | So, try to make it like really just effortless for them in the way that it's like, "Yeah, I get it. I totally get it. I may not do the same things you do, but I understand that impulse to sort of just want more and more and more," and really connecting that to kind of like, "What are your treatment goals? What do you want to do? Is this way effective for |

Notes. SAMHSA guidelines link: Harm Reduction Framework; PWUD = person who uses drugs ; 'overall safety' combines the following codes: safer settings, safer practices, and promote safety ; 'Sustainable workforce, field and infrastructure' combines sustainable workforce and field and sustainable infrastructure' ; dropped 'engage first' as it was not relevant to our project; 'whole person healthcare access' combines the following codes: Provide many pathways to well-being across the continuum of health and social care, safer transitions to care and safer access to healthcare.

| Code Name | SAMHSA Definition | Contextual Definition | Examples |
|---|---|---|---|
| ***Theme 1: The Shifting Landscape of Harm Reduction*** | | | |
| **Sub Theme 1: Changes in Clinical Practice** | | | |
| | the listener's inherent biases, with the intent to fully absorb and process what the speaker is saying. | absorbing and processing what the patient is saying their needs are. | you to achieve the treatment goal that you have set?" I think people enjoy that level of transparency, because it's like, "Oh, you're not telling me what to do, but yeah, you're right. This is really not working too well for me." |
| **Theme 2: Challenges Remain** | | | |
| **Sub Theme 1: Ambivalence towards harm reduction** | | | |
| Ambivalence | | Discussion of clinicians questioning or struggling with harm reduction principles, specifically whether they improve people's lives or the clinician's ability to take care of them or how it influences the clinicians level of accountability | |
| **Sub Theme 2: Focus on Abstinence** | | | |
| Abstinence | | Discussions about 'abstinence' as preferred method and/or belief of what works best for patients | |
| **Theme 3: Supports Needed** | | | |
| **Theme 1: Overall Safety** | | | |
| Overall Safety | Active promotion of safety as defined by the people they serve; impact that law enforcement can have; Education and support describing how to | Overall safety includes discussions about and providing education about risk reduction; risk reduction supplies and materials; safe | It's tough when you try to educate them, and you try to tell them. And a lot of them are saying, "No. I know where I buy my stuff from. I'm sure it's safe." And I've had a few that have overdosed and come |
| Notes. SAMHSA guidelines link: Harm Reduction Framework; PWUD = person who uses drugs ; 'overall safety' combines the following codes: safer settings, safer practices, and promote safety ; 'Sustainable workforce, field and infrastructure' combines sustainable workforce and field and sustainable infrastructure' ; dropped 'engage first' as it was not relevant to our project; 'whole person healthcare access' combines the following codes: Provide many pathways to well-being across the continuum of health and social care, safer transitions to care and safer access to healthcare. | | | |

| Code Name | SAMHSA Definition | Contextual Definition | Examples |
|---|---|---|---|
| ***Theme 1: The Shifting Landscape of Harm Reduction*** | | | |
| **Sub Theme 1: Changes in Clinical Practice** | | | |
| | reduce risk; provision of risk reduction supplies and materials (needle exchange programs; test strips; access to safe environments to live and use | living and use environments; reproductive health education, services and supplies; strategies to promote safety, including law enforcement impact | back. Luckily, they come back with the Narcan. But it's scary the amount of clients that are not – they're really not safe. And these are clients that buy their drugs from the same dealer. So, it's not safe. |
| **Theme 2: Work towards system change** | | | |
| Work toward systems change | Harm reduction approaches, initiatives, programs, and services recognize that trauma; social determinants of health, such as access to healthcare, housing, and employment; inequitable policies; lack of prevention and early intervention strategies; and social support have all had a responsibility in systemic harm. | Discussion about the recognition of how trauma, social determinants of health (e.g., access to healthcare, housing, employment), inequitable policies, offering of harm reduction services based upon current use of different drugs, lack of prevention and early intervention strategies, and social support have all had a responsibility in systemic harm. | As a clinic, we kind of do what I just explained, in that one center with the client. But when it comes to anything with opiates, we're a little bit stricter, because of the risk. Especially with all the fentanyl out there. We're a little bit more flexible with other things, but I wanna say with opiates we kinda have a united front where we're less tolerant of the harm reduction. |
| **Theme 3: Sustainable workforce, field and infrastructure** | | | |
| Sustainable workforce, field and infrastructure | Resources for building and maintaining a skilled, well-supported, and appropriately managed workforce and for sustaining community-based programs (Organizational leadership from people with lived experience; Living wages and essential benefits for harm reduction workers; | Discussion about strategies and resources available to build and maintain a skilled, well-supported, and appropriately managed workforce and community engaged infrastructure (e.g., organizational leadership, staff and peer support include | Right now, we're actually in the process of hiring on a harm reduction specialist specifically for outreach to try to bridge that gap and make it a little bit easier to access them. And also, make it so that when they're ready to actually stop they have an easy pathway back into treatment. |
| Notes. SAMHSA guidelines link: Harm Reduction Framework; PWUD = person who uses drugs ; 'overall safety' combines the following codes: safer settings, safer practices, and promote safety ; 'Sustainable workforce, field and infrastructure' combines sustainable workforce and field and sustainable infrastructure' ; dropped 'engage first' as it was not relevant to our project; 'whole person healthcare access' combines the following codes: Provide many pathways to well-being across the continuum of health and social care, safer transitions to care and safer access to healthcare. | | | |

| Code Name | SAMHSA Definition | Contextual Definition | Examples |
|---|---|---|---|
| ***Theme 1: The Shifting Landscape of Harm Reduction*** | | | |
| **Sub Theme 1: Changes in Clinical Practice** | | | |
| | Training and technical assistance for community-based providers; Co-leadership of PWUD in organizational partnership in research; Promote education on the value of harm reduction services) | people with lived experience, living wages and essential benefits for staff, training and technical assistance for staff and community-based providers). | |
| State agency | | Code for any discussions related to state agency training or regulations, including OASAS | |
| Notes. SAMHSA guidelines link: Harm Reduction Framework; PWUD = person who uses drugs ; ‘overall safety’ combines the following codes: safer settings, safer practices, and promote safety ; ‘Sustainable workforce, field and infrastructure’ combines sustainable workforce and field and sustainable infrastructure’ ; dropped ‘engage first’ as it was not relevant to our project; ‘whole person healthcare access’ combines the following codes: Provide many pathways to well-being across the continuum of health and social care, safer transitions to care and safer access to healthcare. | | | |

## Abbreviations

SUD

substance use disorder

OUD

opioid use disorder

OAT

opioid agonist treatment

NYS

New York State

OASAS

The Office of Addiction Services and Supports

MOUD

medications for opioid use disorder

CARE

Coaching for Addiction Recovery Enhancement

SAMHSA

Substance Abuse and Mental Health Services Administration

ASAM

American Society for Addiction Medicine

### Declarations

**Ethics approval and consent to participate.**

The Institutional Review Board at NYU Grossman School of Medicine reviewed and approved this study.

**Consent for publication.**

Participants agreed to have quotations from their interviews deidentified and used in publication within their consent forms.

## Funding:

1R33DA049252

## Author Contribution

MH coded and analyzed interview transcripts, and was a major contributor in writing the manuscript. MAO conducted interviews, coded and analyzed interview transcripts and was a major contributor in writing the manuscript. SH and AEJ provided revisions on finalized manuscript. CN and PL are MPIs for the overall study. All authors read and approved the final manuscript.

## Acknowledgement not applicable

## Data Availability

The datasets used and/or analyzed during the current study are available from the corresponding author on reasonable request.

### Figures

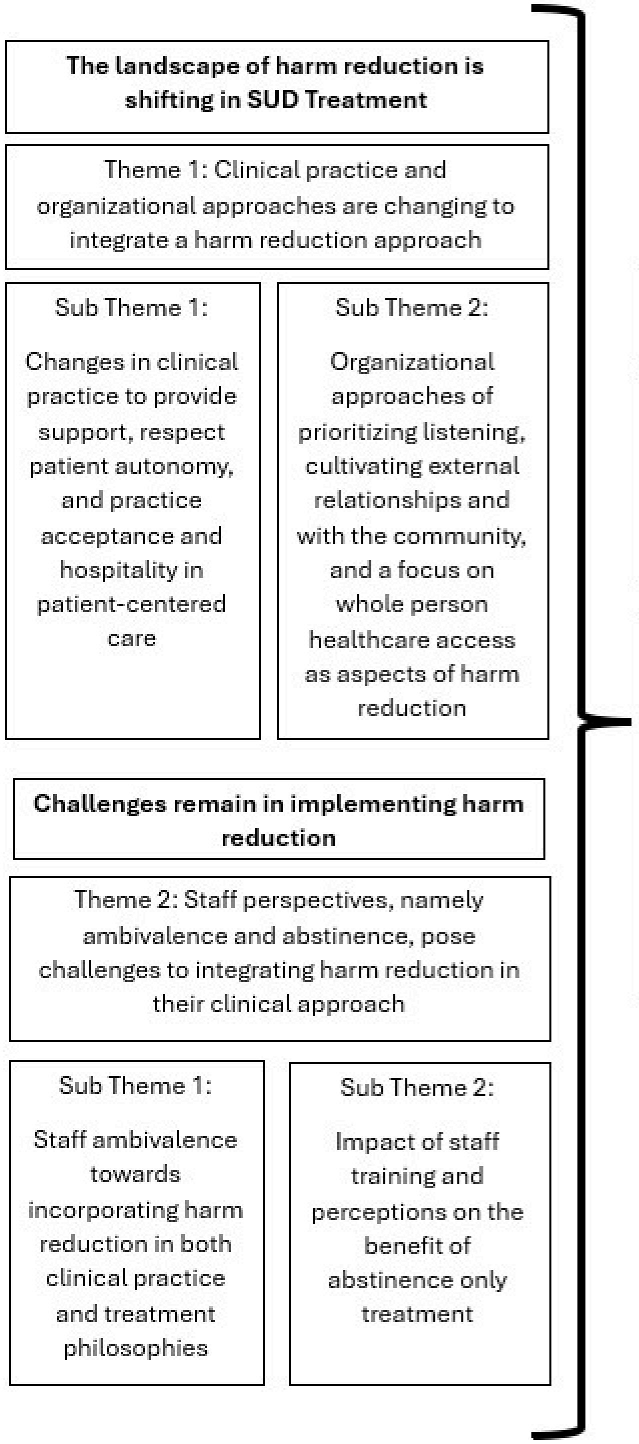

**Figure 1**

Themes and sub-themes
